# Advancing biodiversity assessments with environmental DNA: Long‐read technologies help reveal the drivers of Amazonian fungal diversity

**DOI:** 10.1002/ece3.6477

**Published:** 2020-06-23

**Authors:** Camila D. Ritter, Micah Dunthorn, Sten Anslan, Vitor Xavier de Lima, Leho Tedersoo, Rolf Henrik Nilsson, Alexandre Antonelli

**Affiliations:** ^1^ Eukaryotic Microbiology University of Duisburg‐Essen Essen Germany; ^2^ Gothenburg Global Biodiversity Centre Göteborg Sweden; ^3^ Department of Biological and Environmental Sciences University of Gothenburg Göteborg Sweden; ^4^ Zoological Institute Technische Universität Braunschweig Braunschweig Germany; ^5^ Departamento de Micologia Centro de Biociências Universidade Federal de Pernambuco Recife Brazil; ^6^ Institute of Ecology and Earth Sciences University of Tartu Tartu Estonia; ^7^ Royal Botanic Gardens, Kew Richmond UK

**Keywords:** environmental DNA, high‐throughput sequencing, metabarcoding, neotropical biodiversity, PacBio, third‐generation sequencing

## Abstract

Fungi are a key component of tropical biodiversity. However, due to their inconspicuous and largely subterranean nature, they are usually neglected in biodiversity inventories. The goal of this study was to identify the key determinants of fungal richness, community composition, and turnover in tropical rainforests. We tested specifically for the effect of soil properties, habitat, and locality in Amazonia. For these analyses, we used high‐throughput sequencing data of short and long reads of fungal DNA present in soil and organic litter samples, combining existing and novel genomic data. Habitat type (phytophysiognomy) emerges as the strongest factor explaining fungal community composition. Naturally open areas—campinas—are the richest habitat overall. Soil properties have different effects depending on the soil layer (litter or mineral soil) and the choice of genetic marker. We suggest that campinas could be a neglected hotspot of fungal diversity. An underlying cause for their rich diversity may be the overall low soil fertility, which increases the reliance on biotic interactions essential for nutrient absorption in these environments, notably ectomycorrhizal fungi–plant associations. Our results highlight the advantages of using both short and long DNA reads produced through high‐throughput sequencing to characterize fungal diversity. While short reads can suffice for diversity and community comparison, long reads add taxonomic precision and have the potential to reveal population diversity.

## Graphical abstract

Here we compare the novel long‐read data for metabarcoding with already published short reads to assess Amazonian fungal diversity and community turnover. Habitat type emerges as the strongest factor in explaining fungal community composition. Soil properties have different effects depending on the soil layer (litter or mineral soil) and the choice of genetic marker. Naturally, open areas—campinas—are the richest habitat overall, highlighting the importance of this habitat in microbial diversity.

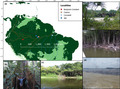

## 1 INTRODUCTION

Fungi are inconspicuous organisms, only a proportion of which sporadically reveal their presence through the formation of tangible morphological structures such as fruiting bodies (Moore, 1985). The study of fungi has therefore benefited immensely from the development of molecular (DNA) sequencing tools during the last 30 years. However, even with the use of molecular tools, studies involving the tropics have neglected fungi, despite the fact that the majority of undescribed fungi are thought to occur in the tropics (Hawksworth, 2001; Hawksworth & Rossman, 1997; Lodge et al., 1995). Among all tropical biomes, rainforests provide the widest range of ecosystem services through high above‐ and below‐ground biodiversity (Wardle et al., 2004), including water cycling and carbon storage (Fearnside, 2008; Ojea, Martin‐Ortega, & Chiabai, 2012). The largest and most diverse of those forests is Amazonia (Antonelli et al., 2018; Hansen et al., 2013), which comprises approximately 40% of the area occupied by rainforest habitats around the world. Amazonian ecosystem services can only be maintained through abiotic and biotic processes, many of which are mediated by fungi.

To better characterize fungal communities in Amazonia, short‐read high‐throughput sequencing (HTS) platforms such as Illumina are being increasingly used (Dunthorn, Kauserud, Bass, Mayor, & Mahé, 2017; Ritter, Zizka, et al., 2019; Ritter et al., 2018; Tedersoo et al., 2014; Vasco‐Palacios, Bahram, Boekhout, & Tedersoo, 2019). These approaches are often used together with PCR techniques to amplify individual markers. In particular, the nuclear ribosomal Internal Transcribed Spacer (ITS) region has been selected as the best DNA region to identify the widest possible range of fungal groups and is therefore commonly used as a universal DNA barcode for fungi (Schoch et al., 2012). This region is typically 500–600 bases long, preventing it from being sequenced under some sequencing technologies. The use of partial sequencing (targeting only a subregion such as ITS1 or ITS2) has at times limited the taxonomic coverage and identification of fungi by not providing enough variation to tell species apart (Nilsson, Ryberg, Abarenkov, Sjökvist, & Kristiansson, 2009). Furthermore, even though HTS approaches produce hundreds of thousands or millions of sequences per sample, the limited length of these sequences can introduce critical biases to the precise taxonomic identification of the underlying lineages (Nilsson et al., 2019; Tedersoo, Tooming‐Klunderud, & Anslan, 2018).

Long‐read HTS has the potential to overcome some of these limitations, but it has rarely been used in environmental studies (Tedersoo et al., 2018; Purahong, Mapook, Wu, & Chen, 2019). One of the most well‐developed platforms is the single‐molecule real‐time sequencing platform of Pacific Biosciences (PacBio^®^) (Rhoads & Au, 2015). Although the PacBio platform had a high error rate at the time it was launched, the error rate is currently less than 1% (Goodwin, McPherson, & McCombie, 2016). Recent studies have shown that the potential of the PacBio platform for the identification of fungal communities using environmental samples is high (Purahong et al., 2019; Tedersoo et al., 2018), but so far it has not been widely applied to any ecosystems.

Taken together, the use of short‐ and long‐sequence HTS techniques offers the potential to overcome the challenges of characterizing fungal diversity in species‐rich ecosystems, such as Amazonia in northern South America. Amazonia is a heterogeneous biome, and its biodiversity has been shown to vary considerably across geographical ranges. On a large scale, a west (more diverse) to east (less diverse) diversity gradient has been observed in many animal and plant groups (Hoorn et al., 2010; Steege et al., 2003; Zizka, ter Steege, Pessoa, & Antonelli, 2018) and also in micro‐organisms, including fungi (Ritter, Faurby, et al., 2019; Ritter, Zizka, et al., 2019). Another source of heterogeneity in Amazonia is the presence of distinct habitats types. Each phytophysiognomy comprises a largely distinct biota, its own soil characteristics, flooding regime, and nutrient availability (Myster, 2016; Ritter et al., 2018). Four widespread and important habitats, here given in the order of decreasing plant and animal diversity (Myster, 2016; Ritter, Faurby, et al., 2019), are as follows: unflooded tropical forests (terra‐firme); forests seasonally flooded by fertile white‐water rivers (várzeas); forests seasonally flooded by unfertile black water rivers (igapós); and naturally open areas associated with white‐sand soils (campinas). The richness gradient for micro‐organisms has been found to differ from this general trend, as campinas harbor the highest microbial richness (Ritter, Faurby, et al., 2019; Ritter, Zizka, et al., 2019).

Soil physicochemical characteristics are often considered crucial for biotic dynamics, vegetation, and diversity patterns at local to regional scales across Amazonia (Higgins et al., 2011; Laurance et al., 2010; Vasco‐Palacios et al., 2019; Vogel et al., 2009). Although several studies have reported on the importance of soil characteristics in shaping community structure, no unified pattern has emerged. In a recent study using HTS with short reads from environmental samples in Amazonia, members of our team showed a mixed effect of soil properties on the microorganism richness and community turnover (Ritter et al., 2018). In that study, we used general primers to target all eukaryotes, and we did not address specifically these effects on fungi.

This study seeks to characterize fungal communities across Amazonia using environmental samples of soil and litter. For the first time (to our knowledge) in an Amazonian context, we use a long‐read approach to sequence the full fungal ITS region on the PacBio platform. In addition, we combine our novel long‐read data with our previously released short‐read HTS data of the nuclear ribosomal 18S rRNA small subunit (18S) gene and the mitochondrial cytochrome c oxidase subunit I (COI) gene produced in an Illumina sequencing platform. We discuss the patterns of fungal richness and community turnover across Amazonia and compare the results obtained from different genes and platforms.

## 2 METHODS

### 2.1 Study area and sampling design

**FIGURE 1:**
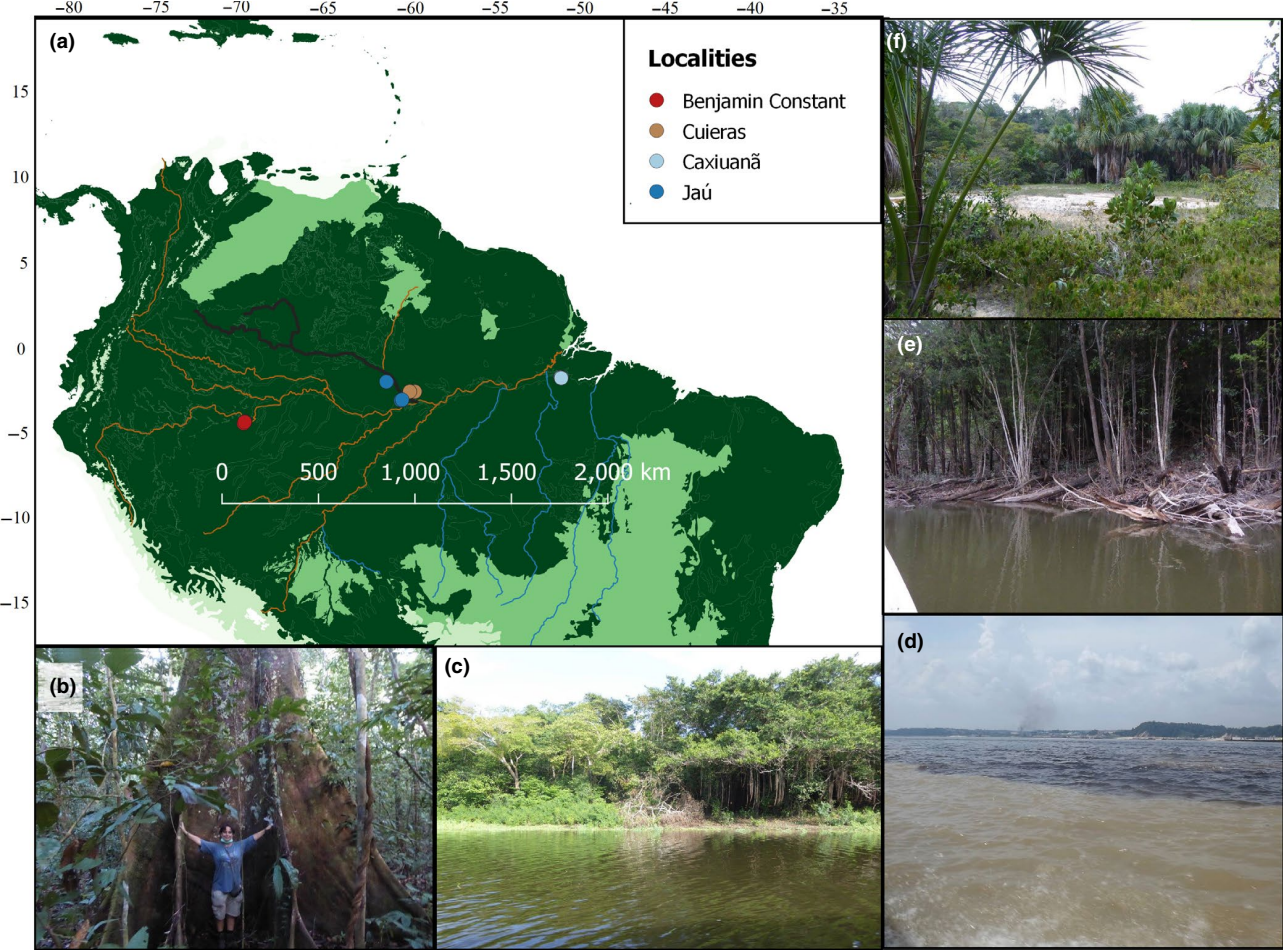
Map of sampling localities and habitats. (a) Northern South America, where dark green represents forest biomes and light green open vegetation biomes, as delimited by Dinerstein et al. (2017). The rivers are colored by the type of water: Brown represents white‐water rivers, black is the Negro river, and blue represents clear water rivers. Circles represent the main localities sampled; (b) Terra‐firme forest with the lead author as size reference; (c) Várzea forest showing the white‐water river; (d) The confluence of the Amazon (white water) and Negro (black water) rivers; (e) Igapó forest showing a black water river; and (f) Campina showing the white sand soil. Map produced in Qgis (Pereira et al., 2019)

We sampled four localities across Brazilian Amazonia (Figure 1) following the sampling design described by Tedersoo et al. (2014). Detailed locality descriptions are available in Ritter, Zizka, et al. (2019). Benjamin Constant (BC), to the south of the Amazon river, is the westernmost study locality (3 igapós, 3 terra‐firme and 3 várzeas plots); Jaú is located to the west and Cuieras to the east of the Negro river, and both are located to the north of the Amazon river (3 campinas, 3 igapós and 3 terra‐firme plots at each); Caxiuanã is located to the south of the Amazon river and is the easternmost study locality (3 campinas, 3 igapós, 3 terra‐firme, and 3várzeas plots). We sampled all depths of the litter layer above the mineral soil (all organic matter, including leaves, roots, and animal debris) and the top 5 cm of the mineral soil in a total of 39 circular plots, each with a radius of 28 m. We chose 20 random trees inside each plot and collected litter and soil on both sides of each tree. We then pooled the samples by substrate to produce one litter sample and one soil sample per plot. The soil physicochemical properties were determined by a Brazilian company (EMBRAPA); additional details of the soil analysis can be found in Ritter et al. (2018).

### 2.2 Data generation

For the nuclear ribosomal small subunit (SSU) 18S rRNA (18S) and the mitochondrial cytochrome c oxidase subunit I (COI) genes, we used the OTU table produced in Ritter, Faurby, et al. (2019). We selected the OTUs assigned to the fungal kingdom based on SILVA (Quast et al., 2012) for 18S and GenBank (Benson et al., 2018) for COI datasets, respectively, for all our analyses. We present here the results of both markers in light of the fact that the previous publication did not analyze fungi separately, which imposed limits on the fungal richness and community structure analyses employed at the time.

For the ITS, we followed the approach described in Tedersoo et al. (2018). We used the forward primers ITS9MUNngs (5′‐TACACACCGCCCGTCG‐3′; Tedersoo & Lindahl, 2016) and ITS4ngsUni (5′‐CCTSCSCTTANTDATATGC‐3′; Tedersoo & Lindahl, 2016) to target the full ITS region (ITS1 ‐ 5.8S ‐ ITS2)". For amplification, we used a PCR mixture comprised 5 μl of The Firepol is a thermostable Taq DNA polymerase used in the amplification. The mix contain the Firepol, primers, DNA and ddH2O, in a total of 25 μl of each forward and reverse primer (20 mM), 1 μl of DNA extract, with the original concentration, and 18 μl ddH_2_O. Thermal cycling included an initial denaturation at 95°C for 15 min; cycles of denaturation for 30 s at 95°C, annealing for 30 s, elongation at 72°C for 1 min; final elongation at 72°C for 10 min and storage at 4°C. The duplicate PCR samples were pooled; their relative quantity was estimated by running 5 μl DNA on 1% agarose gel stained with ethidium bromide (Sigma‐Aldrich, St Louis, MO, USA). We used negative (for DNA extraction and PCR) controls throughout the experiment. The amplicons were purified with FavorPrep PCR Clean Kit (FavorGen Biotech Corporation, Vienna, Austria). The concentration of PCR products was standardized for sequencing. The libraries were prepared using PacBio amplicon library preparation protocol (Pacific Biosciences, Inc) and loaded to seven SMRT cells using the MagBead method. The libraries were sequenced using the PacBio RS II instrument using P6‐C4 chemistry following the manufacturer´s protocol.

Bioinformatics analyses were performed using the PipeCraft platform (Anslan, Bahram, Hiiesalu, & Tedersoo, 2017). PacBio circular consensus reads (CCS, reads_of_insert) were quality filtered with VSEARCH (Rognes, Flouri, Nichols, Quince, & Mahé, 2016) (maxee = 2, maxns = 0, minlen = 150). Filtered reads were demultiplexed based on the unique sequence identifiers using mothur (Schloss et al., 2009) (bdiffs = 1). Putative chimeric reads were filtered using de novo and reference‐database‐based methods in VSEARCH. Additionally, sequences where the full PCR primer was found anywhere in the read were filtered out using the PipeCraft built‐in module, as these reads represent additional chimeras not detected by VSEARCH. The full ITS region was extracted using ITSx (Bengtsson‐Palme et al., 2013) and clustered using the UPARSE algorithm (Edgar, 2013) with a 98% similarity threshold. Additionally, the postclustering curation method LULU (Frøslev et al., 2017) was applied (minimum_ratio_type = “min,” minimum_match = 98) to merge consistently co‐occurring “daughter” OTUs. Taxonomy annotation was performed using BLASTn (Camacho et al., 2009) against the UNITE (Abarenkov et al., 2010; Nilsson et al., 2018) and INSDC (Cochrane, Karsch‐Mizrachi, & Takagi, 2016) databases.

### 2.3 Statistical analysis

We performed all statistical analyses in R v.3.6.0 (R Core Team, 2003). Two samples (SCUICAMP3 and LCUITFP3) had a very low number of reads in the ITS results and were excluded from subsequent analyses of all markers. We use as a diversity estimate the effective number of OTUs, calculated with the unrarefied read counts as OTU abundance, using the exponential of the Shannon entropy diversity of order *q* = 1 (Jost, 2006). This measure is more robust against biases arising from uneven sampling depth than the simple number of OTUs (McMurdie & Holmes, 2014). For the abundance‐based community matrices, we transformed read counts using the “varianceStabilizingTransformation” function in DESeq2 (Love, Huber, & Anders, 2014) as suggested by McMurdie and Holmes (McMurdie & Holmes, 2014). This transformation normalizes the count data with respect to sample size (number of reads in each sample) and variances, based on fitted dispersion–mean relationships (Love et al., 2014).

We tested the correlation between diversity of each marker through a Pearson correlation between each pair of markers. To test between the community composition correlation, we performed a Mantel test with the Jaccard dissimilarity matrices, using the Pearson correlation and 999 permutations for significance. Both analyses were performed using the vegan v.2.5.5 R package (Oksanen et al., 2010).

For soil physicochemical analysis, we first normalized all variables to mean = 0 and variance = 1. We then performed two principal component analyses (PCA), one for soil grain size and the other for chemical compounds, using the vegan package. We used the first axis of each PCA (explaining 56% and 69% of the total variation, respectively) in the subsequent linear models and multiple regressions analysis. Given the expected importance of soil organic carbon content (Nielsen, Ayres, Wall, & Bardgett, 2011; Ritter et al., 2018) and pH (Lauber, Hamady, Knight, & Fierer, 2009; Ritter et al., 2018), we used these as independent variables.

To test the effect of soil properties on fungal OTU richness, we performed a Bayesian general linear model (GLM) analysis, as implemented in the R‐INLA v.17.6.20 R package (Rue et al., 2009). The response variables were the OTU diversity by soil layer (litter and soils) and marker (18S, ITS and COI), giving a total of six models. In each case, the soil properties (PC1 for the physical, PC1 for the chemical, organic carbon content, and pH both standardized to mean = 0 and variance = 1) were used as explanatory variables. We tested the effect of spatial autocorrelation by comparing analyses of standard GLMs with GLM analysis using stochastic partial differential equations (SPDE) that explicitly consider spatial correlation.

To test the effect of soil properties on fungal community turnover, we used multiple regressions on dissimilarity matrices (MRM) with the R package ecodist v.2.0.1 (Goslee & Urban, 2007). The response variables were dissimilarity matrices calculated using the Jaccard dissimilarity. In each case, the explanatory variables were the distance matrices based on soil properties (physical PC1, chemical PC1, organic carbon, and pH) and one geographical distance matrix (all calculated using Euclidean distances). Statistical significance of the regression coefficients was determined using 10,000 permutations.

For the analysis of differences of community composition by locality and habitat, we performed a nonmetric multi‐dimensional scaling (NMDS) analysis using the Jaccard dissimilarity matrix and tested the significance of groups using the envfit test, which fits vectors of continuous variables—in this case the NMDS axes—and centroids of levels of class variables (locality, habitat, and soil layer) using the vegan package. Additionally, we performed a permutational analysis of variance (PERMANOVA) to test the significance of each factor (locality, habitat, soil layer, first PC of both PCAs, pH, and carbon) in the community composition of each dataset (18S, COI, and ITS) using the vegan package. To assess the difference between the habitats and localities, we performed a post hoc test of PERMANOVA using the R package pairwiseAdonis v.0.4 (Arbizu, 2020).

Based on literature and experience, V.X.L. assigned all OTUs classified as fungi to putative functional groups. Based on the literature, OTUs were assigned individually to one of five functional groups: “lichen,” “mycorrhizae,” “parasite,” “phytopathogen,” and “saprobe.” As the name implies, all lichenized fungi are classified as “lichen” (e.g., *Lecanora*, *Lepidostroma*). “Mycorrhiza” are all fungi in a mutualist association with root plants (e.g., Glomeromycotina, *Amanita*). “Phytopathogen” refers to all fungi associated with plant diseases (e.g., *Clodosporium* spp., several Venturiaceae spp.). “Parasite” refers to parasites of other organisms except plants (e.g., most Zoopagomycotina, *Metarhizium* sp.). The fifth category, “Saprobe,” contains all nonobligatory biotrophic fungi, including coprophilous and opportunistic parasites. Most OTUs classified at family level or higher are impossible to categorize confidently (e.g., Agaricales, Dothideomycetes), as are species that are only known from a single or few collections without associated information on their ecology (e.g., *Alloconiothyrium aptrootii*, *Dictyochaeta mimusopis*) were kept as “unknown.” As many OTUs identified at genus level may represent undescribed species, their functional classification was by association. For instance, unidentified species of a genus predominantly composed of saprobe species were also classified as “saprobe” (e.g., *Mucor* spp., *Phlebia* spp.); likewise, those in a predominantly ectomycorrhizal group were classified as “mycorrhiza” (e.g., *Lactarius* spp., *Paxillus* spp.). Nevertheless, several genera are composed of species in two or more functional groups, such as *Bionectria* and *Tricholoma;* in cases such as this, unidentified species were classified as “unknown.”

We performed an analysis of indicator OTUs of each locality, habitat, and soil layer using the R package indicspecies v.1.7.6 (De sCaceres, Jansen, & De Caceres, 2016) using the matrix of relative abundance. This analysis identifies the species, in our case the OTUs, that are associated with a determined group. We performed the analysis three times with each dataset (18S, COI, and ITS): the first grouped the OTUs by locality, the second by habitat, and the third by soil layer. We tested significance with 9,999 permutations, from which we quantified the number of indicator OTUs for each group with an alpha < 0.05. We also used the previous guild classification to categorize all possible indicator OTUs (Table S2).

We calculated the mean number of OTUs by each factor (locality, habitat, and soil layer) in each dataset (18S, COI, and ITS) using the vegan R package. We produced a Venn diagram for visualization of the number and proportion of exclusive and shared OTUs for each factor (locality, habitat, and soil layer) in each dataset (18S, COI, and ITS) using the online tool Venny 2.0 (Oliveros, 2007). Additional R packages used for data curation were tidyverse v.1.2.1 (Wickham, 2017) and ggplot2 v.3.1.1 (Wickham, 2016). All scripts and data used in the analyses are available as supplementary material.

## 3 RESULTS

### 3.1 OTU classification and marker correlation

**FIGURE 2:**
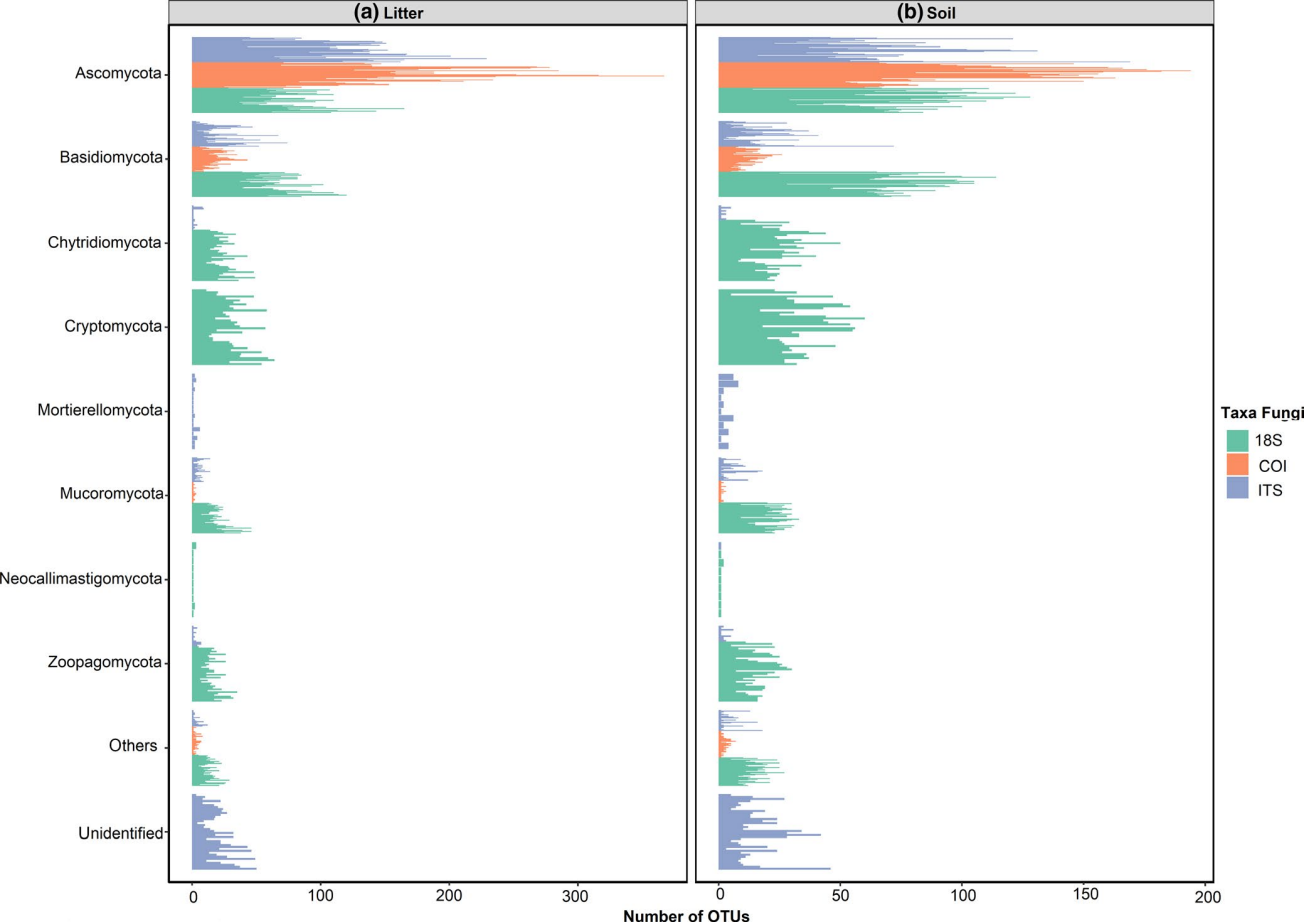
Number of OTUs by fungal phylum. Each bar is the number of OTUs in each plot in (a) litter samples and (b) soil samples. The colors represent the different molecular markers sequenced for this study. All datasets are dominated by Ascomycota, followed by Basidiomycota

After sequencing, processing, and filtering of short reads (Illumina), we found a total of 10,745 OTUs (9,149,502 reads), of which 2,212 (20%) were identified as fungi for the 18S dataset. For COI, we found a total of 6,227 OTUs (242,977 reads), of which 2,161 (35%) were fungal. For the long reads (PacBio) of ITS, we obtained a total of 3,711 OTUs, of which 3,039 (82%) were fungal. The majority of the fungal OTUs were found to belong to the phylum Ascomycota, followed by Basidiomycota (Figure 2). The 18S dataset was found to contain a higher proportion of non‐Dikarya (Ascomycota plus Basidiomycota) than did the other datasets (Figure 2). All the following results are based only on OTUs classified as Fungi.

The effective number of OTUs showed a weak correlation across datasets, with COI being more correlated with 18S (*r* = .36). The ITS was not correlated with either 18S (*r* = −.08) or COI (*r *= −.02). The Mantel tests showed a significant (*p* = .001) correlation in all matrices of similarity, with the strongest correlation between 18S and COI (*r* = .52) and a weaker correlation with the ITS datasets (ITS and COI *r* = .30, ITS and 18S *r* = .17).

### 3.2 Soil characteristics and their effect on fungal diversity and composition

**FIGURE 3:**
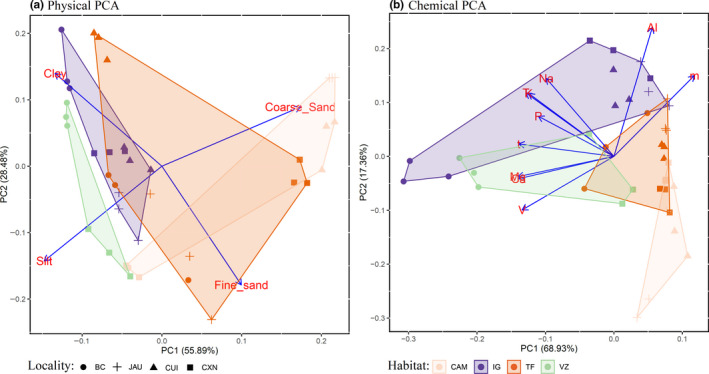
Physical and chemical soil similarity of sample sites across Amazonia. The figure shows the study sites colored by habitat type on the first two axes of a principle component analysis for (a) physical properties (silt, clay, and sand categorized in fine and coarse fractions) and (b) chemical proprieties: phosphorus (P), exchangeable bases (Na, K, Ca, and Mg), exchangeable aluminum (Al), saturation index by aluminum (m), base saturation index (V), effective cation exchange capacity (t), and cation exchange capacity (T). The symbols represent the localities, in the west‐to‐east order: Benjamin Constant (BC), Jaú (JAU), Cuieras (CUI), and Caxiuanã (CXN). The blue rows show the values of each variable's loading in the two first PC axes. For the physical PCA, we found that flooded forests (igapós and várzeas) are associated more fine soil texture (silt and clay), with a wider spread of terra‐firme and campinas. For the chemical PCA, the positive end of the first PC axis, which represents low‐fertility soils, is occupied by a campinas group, followed by terra‐firmes

The principal component analysis (PCA) recovered more than 56% of data variability in the first principal component axis (PC1) for both physical and chemical properties. The PC1 of each PCA was used in further analyses (Figure 3). In our PCA for physical characteristics, the negative values represent fine texture soils (silt and clay), which are predominantly present in seasonally flooded forests—igapós and várzeas (Figure 3a). The campinas had plots at both extremes of PC1, having the plots in Jaú and Cuieras localities with fine texture and the others plots localized in Caxiuanã with coarse soil textures (Figure 3a). Terra‐firme was more spread across different gradients of the soil texture (Figure 3a). In the PCA for chemical compounds, positive values in PC1 represent low‐fertility soils. Campina and terra‐firme were more associated with low‐fertility soils, while várzea forests showed different fertility levels (Figure 3b). Plots in igapó forests also showed low soil fertility except for the plots in Benjamin Constant (Figure 3b). For details of soil characteristics, see Ritter et al. (2018).

Only the mineral soil had some soil properties with a significant effect on the OTU Shannon diversity, an effect that varied by marker (Table 1). For 18S, only the organic carbon (C) content was significant, with a negative effect. Organic carbon was also significant and negative for soil ITS diversity. Chemical PC1 was significant for COI and ITS soil diversity, with a higher effective number of OTUs increase following decreasing soil fertility. The pH and soil texture had no significant effect on OTU diversity.

**TABLE 1:** Soil effects on OTU Shannon diversity by marker

Marker	Soil layer	Variable	Mean	*SD*	0.025	0.5	0.975
18S	Litter	Intercept	3.871	11.000	−19.413	4.034	25.957
		pH	0.067	0.061	−0.054	0.067	0.187
		Carbon	−0.057	0.077	−0.209	−0.056	0.089
		Chemical	0.109	0.090	−0.069	0.109	0.278
		Physical	0.017	0.043	−0.070	0.018	0.100
	Soil	Intercept	3.393	0.703	1.804	3.456	4.653
		pH	−0.019	0.045	−0.108	−0.019	0.070
		Carbon	**−0.287**	**0.048**	**−0.384**	**−0.287**	**−0.194**
		Chemical	0.049	0.034	−0.011	0.048	0.122
		Physical	0.029	0.031	−0.033	0.030	0.089
COI	Litter	Intercept	3.871	11.000	−19.413	4.034	25.957
		pH	0.067	0.061	−0.054	0.067	0.187
		Carbon	−0.057	0.077	−0.209	−0.056	0.089
		Chemical	0.109	0.090	−0.069	0.109	0.278
		Physical	0.017	0.043	−0.070	0.018	0.100
	Soil	Intercept	−1.670	12.588	−26.767	−1.834	24.665
		pH	0.085	0.055	−0.022	0.085	0.192
		Carbon	0.109	0.074	−0.037	0.109	0.253
		Chemical	**0.620**	**0.082**	**0.460**	**0.619**	**0.782**
		Physical	−0.019	0.035	−0.087	−0.019	0.050
ITS	Litter	Intercept	3.871	11.000	−19.413	4.034	25.957
		pH	0.067	0.061	−0.054	0.067	0.187
		Carbon	−0.057	0.077	−0.209	−0.056	0.089
		Chemical	0.109	0.090	−0.069	0.109	0.278
		Physical	0.017	0.043	−0.070	0.018	0.100
	Soil	Intercept	−1.400	10.548	−22.631	−1.470	20.545
		pH	−0.114	0.058	−0.229	−0.114	0.000
		Carbon	**−0.389**	**0.085**	**−0.557**	**−0.388**	**−0.224**
		Chemical	**0.319**	**0.081**	**0.161**	**0.319**	**0.480**
		Physical	−0.046	0.037	−0.119	−0.046	0.027

Note

The table shows the coefficients of each predictor in four Bayesian general multivariate regression models using stochastic partial differential equations (SPDE) that explicitly consider spatial correlation, modeling OTU diversity dependent on soil properties for Amazonian fungi in litter and soil. Since the organic carbon content and pH are considered important variables for soil biota, we use them as independent variables. Bold indicates important predictor variables (credible intervals not crossing zero). The importance of soil properties differed between markers and were significant only for the soil diversity. Carbon content was important for 18S and ITS soil, and chemical PC1 was important for COI and ITS.

Geographical distance was significant for all datasets. However, since juxtaposed localities are usually similar in many respects, we cannot differentiate the level of spatial correlation from the effect of soil properties in our analysis of community turnover (Table 2). For community turnover, organic carbon and pH were significant for all soil communities (18S, COI and ITS), as was pH for all litter communities. Organic carbon was also significant for the COI litter dataset. Soil texture was significant in all communities except for the ITS soil dataset (Table 2). The PC1 for chemical properties was significant for the 18S and COI litter communities. In the PERMANOVA analysis, the soil properties were all significant with a low effect on all datasets (Table S3).

**TABLE 2:** Association between environmental distance and community turnover

Marker	Predictor	Litter		Soil	
		Coefficients	*p* value	Coefficients	*p* value
18S	Intercept	94.615	1.000	77.103	1.000
	Geo.Dist	**0.144**	**.003**	**0.084**	**.050**
	pH	**0.193**	**.002**	**0.143**	**.026**
	Carbon	0.110	.096	**0.286**	**.001**
	Chemical	**0.168**	**.015**	0.109	.162
	Physical	**0.115**	**.035**	**0.160**	**.017**
COI	Intercept	18.726	1.000	−1.402	1.000
	Geo.Dist	**0.114**	**.007**	**0.192**	**.000**
	pH	**0.175**	**.008**	**0.130**	**.030**
	Carbon	**0.267**	**.001**	**0.299**	**.000**
	Chemical	**0.177**	**.023**	0.137	.069
	Physical	**0.215**	**.002**	**0.246**	**.000**
ITS	Intercept	157.504	1.000	110.212	1.000
	Geo.Dist	**0.116**	**.015**	**0.094**	**.033**
	pH	**0.229**	**.006**	**0.180**	**.010**
	Carbon	0.111	.223	**0.362**	**.000**
	Chemical	−0.115	.227	0.046	.589
	Physical	**0.212**	**.006**	0.005	.945

Note

The multiple regressions were based on the geographical distance, Euclidean distance matrices of soil properties, and community Jaccard dissimilarity index values. Bold indicates significant results. Community dissimilarity is significantly associated with geographical distance (Geo.Dist) for Amazonian fungal communities in soil and litter. All community turnovers were significant using 10,000 permutations (*p* < .05) with the following *R* ^2^: 18S litter = .18 (*F* = 31.6) and soil = .18 (*F* = 30.1), COI litter = .26 (*F* = 50.2) and soil = .28 (*F* = 54.5), and ITS litter = 0.12 (*F* = 18.8) and soil = .18 (*F* = 30.1).

**FIGURE 4:**
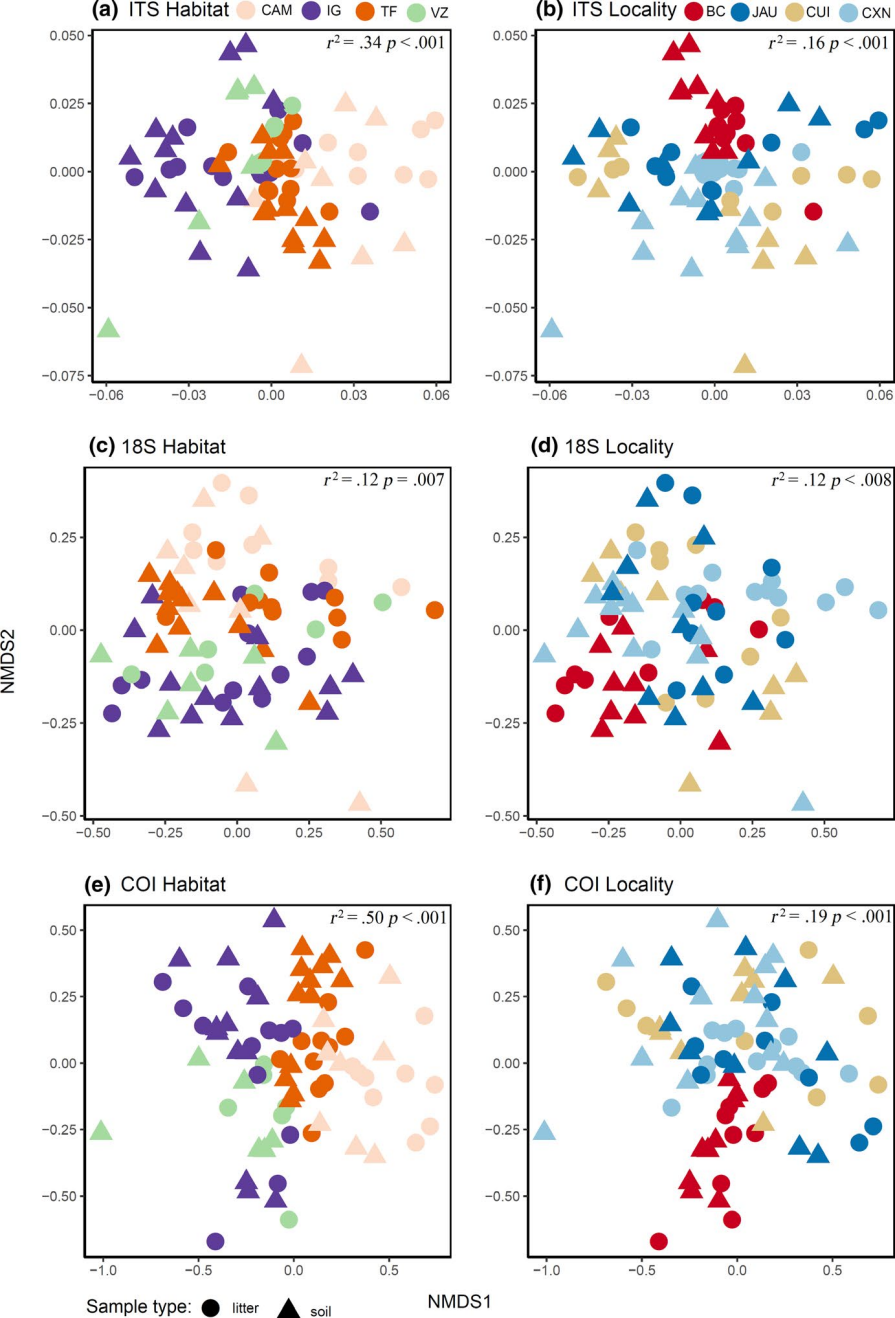
Community structure related to substrate type (litter and soil), locality, and habitat type. Visualization of differences in OTU composition (assessed through abundance matrices using the Jaccard dissimilarity index) using nonmetric multi‐dimensional scaling (NMDS) for (a) ITS by habitat, (b) ITS by locality, (c) 18S by habitat, (d) 18S by locality, (e) COI by habitat, and (f) COI by locality. Circles represent litter samples and triangles soil samples. Both the habitat and the locality factor were statistically significant (EnvFit test). The *R* ^2^ and *p* values of each test are provided inside each subfigure. The strongest and most significant separation is observed between habitat types

**FIGURE 5:**
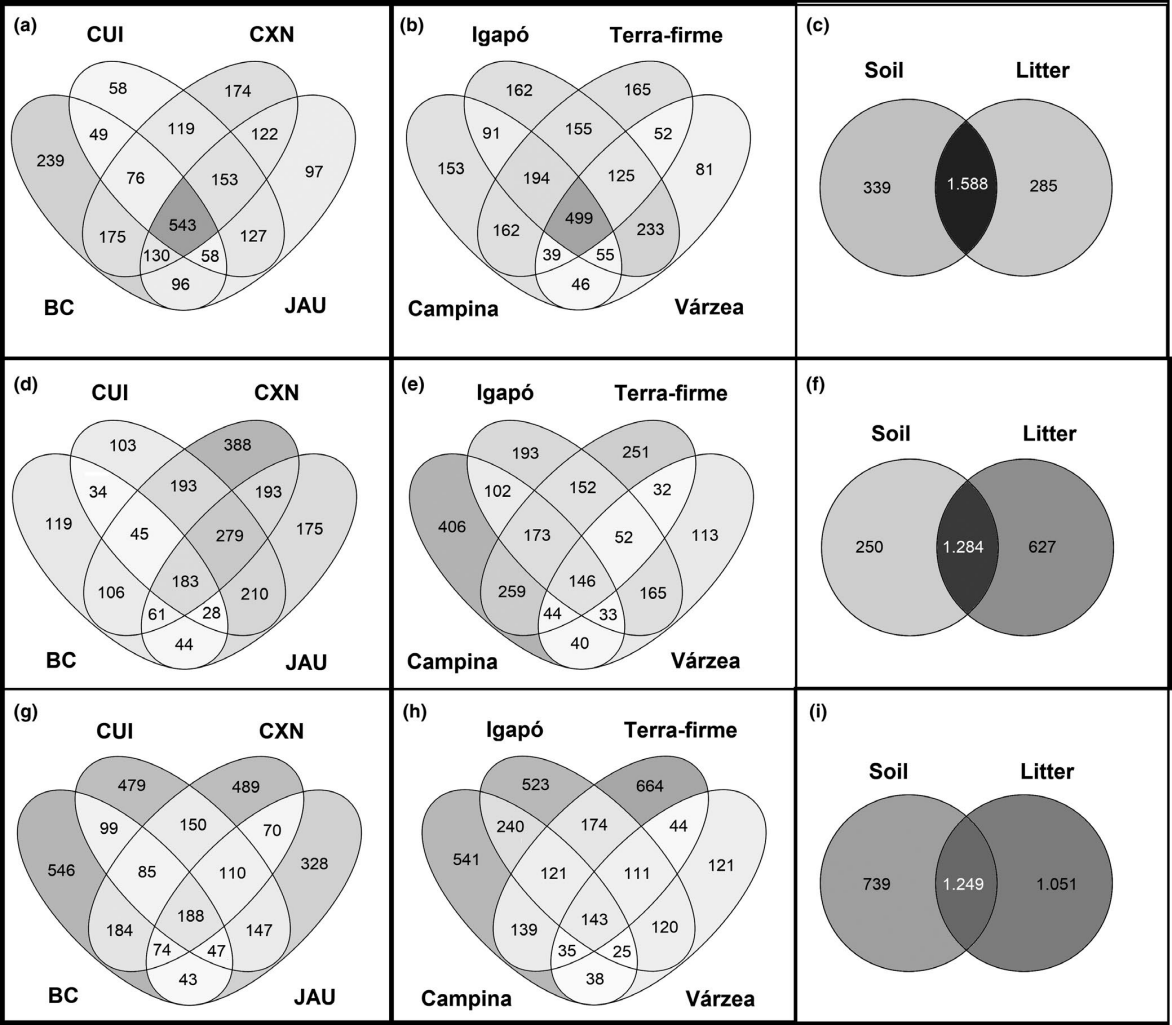
Venn diagrams showing the number of exclusive and shared OTUs for localities (a), habitats (b), and sample type (c) in the 18S dataset; for localities (d), habitats (e), and sample type (f) in the COI dataset; and for localities (g), habitats (h), and sample type (i) in the ITS dataset

The soil layer, organic litter, and mineral soil had a low but significant effect on the number of OTUs (PERMANOVA results: *p* < .001 for all datasets, 18S – *R* ^2^ = .05, COI – *R* ^2^ = .04, and ITS – *R* ^2^ = .03). There were small differences between the soil and litter communities in the two axes of nonmetric multi‐dimensional scaling (NMDS) in all datasets (Figure 4). The litter COI and ITS datasets had a higher mean number of OTUs, where a higher number of OTUs is considered litter indicators (OTUs with a significantly higher probability to be found in litter than soil; Table 3), and a high number of exclusive OTUs than 18S (Figure 5). For 18S, the results contrast with those of the other markers, showing soil as the most diverse substrate, with the highest number of exclusive and indicator OTUs (Table 3, Figure 5c). The majority of indicator OTUs for both layers are saprotrophs (Table S2).

**TABLE 3:** Mean number of OTUs and number of indicator OTUs of Amazonian fungi by markers in each locality, habitat, and soil layer

		18S		COI		ITS	
		Mean	Indicator	Mean	Indicator	Mean	Indicator
Locality	BC	**436**	**90**	107	75	**165**	174
	JAU	369	73	176	98	111	43
	CUI	338	58	181	**173**	142	**189**
	CXN	386	52	**222**	153	148	58
Habitat	TF	376	36	179	108	139	58
	VZ	399	101	145	184	127	118
	IG	370	61	133	79	144	73
	CAM	**404**	**173**	**252**	**358**	**156**	**144**
Soil layer	Litter	375	42	**209**	**169**	**176**	**98**
	Soil	**393**	**106**	142	29	109	12

Note

Localities are ordered west to east: BC = Benjamin Constant, JAU = Jaú, CUI = Cuieras, and CXN = Caxiuanã. Habitats are ordered by plant and vertebrate diversity gradient: TF = Terra‐firme, VZ = Várzea, IG = Igapó, and CAM = Campina. The highest number in each group is given in bold. Although the richest locality and soil layer varies depending on marker, for habitats campinas are consistently the richest for all markers.

### 3.3 The effect of localities

Regarding locality, Benjamin Constant had the most differentiated community in all datasets (Figure 4). The effect of localities was significant (*p* < .001) and had a higher effect than the soil layer factor in explaining the community composition in all datasets (18S – *R* ^2^ = .10, COI – *R* ^2^ = .12, and ITS – *R* ^2^ = .11). Benjamin Constant was significantly different from all other localities for all datasets (Table S4). In general, the pattern of highest mean, number of exclusive, and number of indicator OTUs by locality varied between markers (Table 3, Figure 5). For 18S and ITS, Benjamin Constant had the highest mean number of OTUs (Table 3) and the highest number of exclusive OTUs (Figure 5). Benjamin Constant also had the highest number of OTUs considered indicators of this locality for the 18S dataset (Table 3). Cuieras had the lowest number of exclusive OTUs for COI and 18S but had the highest number of indicator OTUs for the COI datasets (Figure 5). The majority of indicator OTUs at all localities were saprotrophs, followed by a high proportion of OTUs that could not be classified by their functional group (Table S2).

### 3.4 The effect of habitat

Habitat type was the strongest factor (*p* < .002) explaining community composition in the PERMANOVA analysis (18S – *R* ^2^ = .12, COI – *R* ^2^ = .18, and ITS – *R* ^2^ = .08), with the exception of the ITS dataset. All habitats were significantly different from each other in the 18S and COI datasets (Table S4). For ITS, campinas were significantly different from terra‐firme and igapós, and igapós were also significantly different from várzeas (Table S4). In NMDS, the seasonally flooded forests, igapós, and várzeas were more similar to each other than to campinas and terra‐firmes, which were the most similar to each other (Figure 5). Campinas had the highest mean number of OTUs and the highest number of OTUs considered indicators of this habitat for all datasets (Table 3). Regarding the number of exclusive OTUs, campinas had the highest number of OTUs in the COI datasets (Figure 5e). Terra‐firme was the habitat with the highest number of exclusive OTUs for the 18S and ITS datasets (Figure 5b,h). In all habitats, the majority of indicator OTUs were saprotrophs, followed by a high proportion of OTUs that could not be classified by their functional group (Table S2). The functional guilds by habitat and their proportion are shown in Table S5.

## 4 DISCUSSION

Our results highlight the importance of habitat type for fungal community composition in Amazonia and suggest that Amazonian fungi have different diversity patterns for habitat and locality variables, with the importance of each predictor varying between markers. By contrast, community turnover shows a consistent pattern, with habitat being a strong factor explaining community similarity between plots. This is likely to be because different areas can have similar species richness but different species composition, due to historical, geographic, and environmental factors. For instance, in a study of leaf litter fungi in Central Amazonia, the abundance and richness of fungal morphospecies did not change between low and high rainfall periods, but there was a low proportion of shared morphospecies between periods (Braga‐Neto, Luizão, Magnusson, Zuquim, & de Castilho, 2008). Our results also showed a low proportion of shared OTUs when compared with a HTS study of micro‐organisms in general in the same area (Ritter, Zizka, et al., 2019) (Figure 5).

Soil texture did not explain fungal diversity, while chemical soil characteristics were of importance for COI and ITS soil communities, indicating a high diversity in less fertile soil (Table 1). Although it appears counter‐intuitive, the habitat with lowest soil fertility was the one with highest fungal and other microbial diversity: the campinas (Ritter, Faurby, et al., 2019; Ritter, Zizka, et al., 2019). These results suggest that factors other than soil properties explain a habitat's fungal diversity and community composition.

The soil diversity of the 18S dataset was negatively correlated with carbon, while the specifics of the other datasets were not related to carbon. This could be explained by taxonomic coverage of the 18S dataset, which included the Chytridiomycota and Mucorales (mostly comprising saprotrophic fungi) (Barr, 2001; Benny, Humber, & Voigt, 2014). Saprobes decompose matter into various constituent components, making the nutrients available to other organisms. Saprobes are, in other words, important agents in carbon cycling (Swift, 1982). Hence, a high fungal richness may lead to a faster carbon decomposition in soil, as well as a faster carbon assimilation in the above‐ground biomass. This is in agreement with Liu et al. (2015), who found that phylotype richness and phylogenetic diversity of black soil fungi responded negatively to total carbon content in China. Experiments controlling the variables and quantifying the above‐ground biomass are necessary to further verify these observations.

Contrary to our expectations, pH had no effect on fungal richness. This finding was surprising, since soils with more neutral pH generally have a higher richness of micro‐organisms (Glassman, Wang, & Bruns, 2017; Ritter et al., 2018; Rousk et al., 2010; Wang et al., 2015). Our soil samples were all acidic, with the pH varying between 3.5 and 5.14. Soil fungi studied by Liu et al. (2015) displayed a similar pattern to that reported in this study—a higher relative influence of soil carbon content than of soil pH. They also noted that fungi often have a wider tolerance to pH variation than other micro‐organisms, suggesting that in soils with low pH variation such as presented here, the acidity impact should be less striking (Liu et al., 2015). On the other hand, pH was important in explaining community turnover for all datasets (Table 2). Furthermore, in tropical areas the relationship between fungal communities and soil pH is affected by the fungal trophic guilds (Pärtel, Bennett, & Zobel, 2016). It may indicate that in a highly diverse area, such as Amazonia, fungal diversity will not be impacted by pH variation but there will be a turnover of fungal species related to the pH range.

### 4.1 Spatial differences

Different Amazonian habitats varied considerably in their biotic composition (Borges et al., 2016; Ritter, Zizka, et al., 2019). Habitat was the most significant factor explaining community turnover in 18S and COI datasets. All habitats were significantly different from each other in the 18S and COI datasets (Table S4). In the ITS data, we found that campinas and igapós are dissimilar in their communities (Figure 4a). This can be explained by the physicochemical soil properties (Figure 3). When it comes to chemical properties, campinas and igapós were placed at opposite extremes of PC1 and PC2 (Figure 3b). With respect to the physical properties, campinas have plots in both extremes of PC1, but igapós were better explained by clay content (Figure 3a). Clay content was an important factor in explaining leaf litter in central Amazon fungi (Braga‐Neto et al., 2008). Campinas communities were also significantly different from terra‐firme and igapós, and várzeas were dissimilar in their communities (Figure 4a, Table S4). However, in contrast with campinas and igapós, these differences cannot be explained by soil properties and may be more related to the difference in plant communities (Peay, Baraloto, & Fine, 2013).

For the 18S and COI data, the similarity between habitats is better explained by comparing seasonally flooded and nonflooded habitats (Figure 4). In both communities, igapós and várzeas are similar to each other and distinct from terra‐firme and campinas. This is in agreement with results from studies of micro‐organisms in general in the same areas (Ritter, Zizka, et al., 2019). These results were expected, as the flooded period is a powerful factor that selects for a very specific vegetation type (Assis et al., 2015; Haugaasen & Peres, 2006; Myster, 2016; Steege & Hammond, 2001). Igapós and várzeas are more restricted to a fine soil texture, while in terra‐firme and campinas the soil texture varies more (Figure 3a). However, regarding the chemical properties, terra‐firme and campinas have almost exclusively poor soils, while igapós and várzeas present different gradients of soil fertility (Figure 3b). These distinct patterns among markers might be explained by the differences in taxonomic coverage of each marker, since different species of fungi have distinct habitat preferences (Tedersoo et al., 2014).

We were surprised to find that campinas were, on average, the richest habitat for fungi. This stands in contrast to patterns observed for animals and plants (Adeney, Christensen, Vicentini, & Cohn‐Haft, 2016; Damasco, Vicentini, Castilho, Pimentel, & Nascimento, 2013), and fungi in Colombian Amazonia (Vasco‐Palacios et al., 2019). One explanation for the campinas being the richest environment may be the need for plants to associate with micro‐organisms that fix nutrients in the poor soil habitats. For instance, some studies of campinas in Amazonia address the diversity of ectomycorrhizal fungi (Roy et al., 2016; Singer & Aguiar, 1986; Singer & Araujo, 1979; Singer, Araujo, & Ivory, 1983; Vasco‐Palacios, Hernandez, Peñuela‐Mora, Franco‐Molano, & Boekhout, 2018). The general pattern is that the diversity of ectomycorrhizal fungal diversity is the highest in temperate zones (Tedersoo et al., 2012, 2014; Tedersoo & Nara, 2010), but due to the poor soil in campinas, the ectomycorrhizal fungi will be more diverse.

The origin of the campinas environments in Amazonia is debated (Adeney et al., 2016), but the nature of their soil, which is characterized by high drainage and high acidity, is considered one of the poorest in the world (Janzen, 1974). In this context, Singer et al. (1983) hypothesized that the ectomycorrhizal fungi increase the ability of their host plant to acquire nutrients and water in these very stressful habitats. We found a high richness and number of indicator OTUs in campinas (Figure 5, Table 3), suggesting that the campinas may be hotspots for the diversity of fungi and other micro‐organisms. However, we detected very few mycorrhizal indicator OTUs, although these results could be biased by the lack of representative DNA sequences from tropical areas (Looney, Ryberg, Hampe, Sánchez‐García, & Matheny, 2016)—the high number of unclassified guilds supports this (Table S5). The most up to date list of tropical ectomycorrhizal fungi includes just 135 species (http://tropicalfungi.org/wp‐content/uploads/UPDATED‐Total‐Taxa‐List‐12‐25‐17.pdf) and most of them are not from campinas studies (Roy et al., 2016). In another study, 15 ectomycorrhizal fungi species were found in campinas based on ITS sequencing (Vasco‐Palacios et al., 2018). However, these studies sampled only ectomycorrhizal host trees, which optimizes the detection of ectomycorrhizal fungi. It is interesting that várzea areas have fewer OTUs that correspond to known mycorrhizal species for the three markers. Of the four habitats analyzed, várzea soils exhibit the highest fertility as they are flooded by nutrient‐rich waters, decreasing the necessity for plants to associate with mycorrhizal fungi, in accordance with the hypothesis proposed by Singer et al. (1983).

### 4.2 Comparison between short and long reads and markers

Our results showed a similar pattern for the habitat diversity of long and short reads, corroborating the patterns previous reported (Ritter, Faurby, et al., 2019; Ritter, Zizka, et al., 2019; Ritter et al., 2018). These similarities support the view that our findings are real and independent of any possible methodological biases introduced by the different markers and platforms.

The importance of soil properties on the diversity and community turnover varied among markers. We acknowledge the different taxonomic coverages of each marker and the limitations of the available databases. For instance, the diversity of the early‐diverging fungal lineages Chytridiomycota, Cryptomycota, and Zoopagomycota using 18S is higher and it is in stark contrast with the ITS and COI data. Also, Mortierellomycotina were only detected with ITS. This difference may be the result of either PCR biases and primer choices that amplify some groups better than others, or of gaps in the reference databases used. The ITS and 18S reference databases are well populated for fungi, but due to the most universal coverage of 18S, some groups were more detected but not the Mortierellomycotina that was able to be detected with the ITS primers. The COI is usually used as barcode for metazoans (Huang, Meier, Todd, & Chou, 2008), with lower sequence available for fungi. Our COI data showed around 40% of unidentified OTUs (Ritter, Faurby, et al., 2019), which could represent at least in part some fungal lineages without public reference sequences. Uneven availability of reference sequences may have had impact on our diversity and community composition results for the various markers used, with the highest effect for the COI results.

The use of short‐read fragments (for both 18S and COI) resulted in a higher number of OTUs, for all organisms, than did the long‐read technique. Long‐read ITS, on the other hand, detected more fungal OTUs even though the total number of OTUs was smaller than for short reads. It is important to stress here that, unlike for the ITS region, for short reads we used general primers targeting all eukaryotes and not just fungi, such that only a portion of reads belonged to fungi in the 18S and COI datasets. In addition, the ITS data did not hit the asymptote for most of the plots (Figure S1) and was worse for soil samples. This result could be explained by the fast DNA degradation in hot and humid environments (Taberlet, Coissac, Hajibabaei, & Rieseberg, 2012), which makes it harder to sequence long‐read DNA fragments, and also the poor read depth of the PacBio platform.

Although the differences in primer design preclude us from reliably identifying the “best” marker or sequencing platform choice for fungal assessments in general, we highlight the main advantages and disadvantages of those used here. On the one hand, we showed that the use of 18S under the Illumina platform provides the overall highest taxonomic coverage (Ritter, Faurby, et al., 2019; Ritter, Zizka, et al., 2019). So for studies aiming to compare diversity and community turnover, the use of short reads can be recommended. In economic terms, this is also currently the more cost‐efficient option. However, due to the short fragment size of Illumina reads, some OTUs could be potentially misidentified or categorized only at, for example, the family or genus level. For instance, in an earlier study comparing the taxonomic identification of short‐read HTS, the choice of the ITS subregion, ITS1 or ITS2, affected 51% of fungal identifications (Nilsson et al., 2009). Long‐read HTS methods have the potential to identify fungi with higher accuracy, despite recording fewer sequences per sample (Tedersoo et al., 2018). In our data, PacBio detected the highest number of OTUs classified as fungi but the lowest number of total OTUs. This is expected, since PacBio platforms have a small number of reads in total (Quail et al., 2012) and also will not sequence partially degraded DNA. Additionally, long reads have the potential of combining population analysis with environmental data. This is limited with short reads, which provide a more limited genetic variation for environmental diversity analysis or require the sequencing of several markers for a limited number of target individuals.

## 5 CONCLUSIONS

Tropical fungal diversity is surprisingly high and poorly understood. In our study, we found that the equivalent to a teaspoon of Amazonian soil contained as many as 1,800 OTUs, of which up to 400 were classified as fungi. It might therefore not be an exaggeration to call fungal diversity the “dark matter” of life on Earth, alongside many other poorly studied groups. Our results highlight the importance of habitat type for fungal community composition. We also show that the known general patterns found for macro‐organisms in Amazonia may not apply to fungi. It is important to improve our understanding of the patterns and drivers of fungal diversity and community composition, since this is one of the most diverse eukaryotic kingdoms, whose members play key roles in nutrient cycling and biotic interactions in terrestrial ecosystems. Deforestation of Amazonia is increasing rapidly (Pereira, Ferreira, de Santana Ribeiro, Carvalho, & de Barros Pereira, 2019), and to protect this vast biome it is fundamental to understand the processes underpinning ecosystem stability. For this, we have to identify and understand the distribution and diversity of organisms essential for ecosystem functionality, including fungi.

## CONFLICT OF INTEREST

The authors declare no conflict of interest.

## AUTHOR CONTRIBUTIONS

**Camila D. Ritter:** Conceptualization (equal); data curation (equal); formal analysis (equal); funding acquisition (equal); investigation (equal); methodology (equal); project administration (equal); supervision (equal); validation (equal); visualization (equal); writing–original draft (equal); writing–review and editing (equal). **Micah Dunthorn:** Investigation (equal); writing–review and editing (equal). **Sten Anslan:** Data curation (equal); formal analysis (equal); methodology (equal); writing–review and editing (equal). **Vitor Xavier de Lima:** Investigation (equal); writing–review and editing (equal). **Leho Tedersoo:** Investigation (equal); methodology (equal); project administration (equal); writing–review and editing (equal). **R. Henrik Nilsson:** Conceptualization (equal); funding acquisition (equal); methodology (equal); writing–review and editing (equal). **Alexandre Antonelli:** Conceptualization (equal); funding acquisition (equal); project administration (equal); writing–review and editing (equal).

## Supporting information

Supplementary Material

Supplementary Material

Supplementary Material

Supplementary Material

Supplementary Material

Supplementary Material

Supplementary Material

Supplementary Material

Supplementary Material

## Data Availability

All raw 18S and COI sequences are available in GenBank under BioProject PRJNA464362. PacBio raw sequences are available in GenBank under BioProject PRJNA627319.
