# Supplementary material for: Advancing biodiversity assessments with environmental DNA: Long‐read technologies help reveal the drivers of Amazonian fungal diversity
Source: Ecol Evol. 2020 Jun 23;10(14):7509–24. doi: 10.1002/ece3.6477 (PMC7391351; doi:10.1002/ece3.6477)
Supplement: Supplementary file 1 — Supplementary Material [file ECE3-10-7509-s001.docx]

**SUPPLEMENTARY MATERIAL FOR:**

**Advancing biodiversity assessments with environmental DNA: Long-read technologies help reveal the drivers of Amazonian fungal diversity**

Camila D. Ritter^1,2,3*^, Micah Dunthorn^1^, Sten Anslan^4^, Vitor X. De Lima^5^, Leho Tedersoo^6^, R. Henrik Nilsson^2,3,§^, Alexandre Antonelli^2,3,7,§^

^1^ Department of Eukaryotic Microbiology, University of Duisburg-Essen, Universitätsstrasse 5 D-45141 Essen, Germany

^2^ Gothenburg Global Biodiversity Centre, Box 461, SE-405 30 Göteborg, Sweden

^3^ Department of Biological and Environmental Sciences, University of Gothenburg, Box 463, SE-405 30 Göteborg, Sweden

^4^ Zoological Institute, Technische Universität Braunschweig, Box: 38092, D-38106, Braunschweig, Germany

^5^ Departamento de Micologia, Centro de Biociências, Universidade Federal de Pernambuco, Av. Avenida da Engenharia s/n, CEP: 2341369, Recife, PE, Brazil

^6^ Institute of Ecology and Earth Sciences, University of Tartu, 40 Lai Street, Tartu 51005, Estonia

^7^ Royal Botanic Gardens, Kew, TW9 3AE, Richmond, Surrey, UK

*Corresponding author: Camila D. Ritter, kmicaduarte@gmail.com. Phone: +55 48991434597. Postal address: University of Duisburg-Essen, Universitätsstrasse 5 - D-45141 Essen, Germany.

^§^Both authors contributed equally to the paper.

**Table S1.** Number of reads, total richness and effective number of OTUs calculated with Shannon entropy in the order of q= 1 estimates for Amazonian fungi for each marker. The plots are labelled by soil layer (L = litter, S = mineral soil), locality (BC = Benjamin Constant, CUI = Cuieras, CXN = Caxiuanã, JAU = Jaú) and habitat (CAM = Campinas, IG = Igapó, TF = Terra-Firme, VZ = Várzea).

|  | **18S** | | | **COI** | | | **ITS** | | |
| --- | --- | --- | --- | --- | --- | --- | --- | --- | --- |
| **Plot** | **Reads** | **Richness** | **effN** | **Reads** | **Richness** | **effN** | **Reads** | **Richness** | **effN** |
| LBCIGP1 | 6255 | 375 | 1.70 | 96672 | 111 | 19.37 | 2375 | 288 | 81.24 |
| LBCIGP2 | 5622 | 226 | 15.78 | 20716 | 27 | 1.39 | 5630 | 212 | 23.80 |
| LBCIGP3 | 26814 | 519 | 20.18 | 105488 | 97 | 32.32 | 1090 | 216 | 131.72 |
| LBCTFP1 | 29780 | 437 | 26.97 | 104511 | 154 | 16.63 | 2473 | 242 | 64.93 |
| LBCTFP2 | 8481 | 273 | 67.94 | 89117 | 193 | 9.56 | 1542 | 196 | 74.35 |
| LBCTFP3 | 12073 | 264 | 23.44 | 80622 | 186 | 26.15 | 4170 | 386 | 123.63 |
| LBCVZP1 | 24686 | 519 | 12.09 | 79916 | 166 | 71.85 | 2543 | 119 | 27.01 |
| LBCVZP2 | 5649 | 261 | 9.00 | 83238 | 131 | 16.29 | 1553 | 151 | 53.96 |
| LBCVZP3 | 13774 | 364 | 2.39 | 95551 | 102 | 14.45 | 1822 | 94 | 20.03 |
| LCUICAMP1 | 10534 | 300 | 36.34 | 65848 | 273 | 63.21 | 2710 | 308 | 31.99 |
| LCUICAMP2 | 20346 | 260 | 25.00 | 78704 | 235 | 34.78 | 937 | 142 | 52.31 |
| LCUICAMP3 | 28881 | 246 | 36.57 | 72979 | 305 | 33.84 | 3595 | 254 | 18.40 |
| LCUIIGP1 | 5035 | 201 | 55.13 | 97850 | 159 | 28.32 | 1634 | 225 | 50.28 |
| LCUIIGP2 | 23429 | 132 | 27.77 | 89731 | 127 | 23.78 | 2336 | 174 | 13.30 |
| LCUIIGP3 | 31459 | 172 | 38.34 | 88110 | 194 | 34.14 | 2338 | 158 | 15.24 |
| LCUITFP1 | 4193 | 163 | 5.92 | 83366 | 171 | 11.81 | 1897 | 144 | 26.30 |
| LCUITFP2 | 23419 | 343 | 76.97 | 82959 | 305 | 100.68 | 2236 | 246 | 25.54 |
| LCXNCAMP1 | 2310 | 183 | 44.63 | 68005 | 446 | 70.82 | 2579 | 172 | 32.87 |
| LCXNCAMP2 | 19877 | 383 | 17.93 | 75237 | 392 | 140.54 | 2459 | 220 | 36.93 |
| LCXNCAMP3 | 2961 | 225 | 14.43 | 44993 | 296 | 34.64 | 2564 | 169 | 60.36 |
| LCXNIGP1 | 15431 | 262 | 20.19 | 55496 | 188 | 26.98 | 1559 | 142 | 37.28 |
| LCXNIGP2 | 16068 | 259 | 30.79 | 69353 | 228 | 9.76 | 9837 | 140 | 95.72 |
| LCXNIGP3 | 9636 | 215 | 8.68 | 47995 | 194 | 29.85 | 3213 | 134 | 75.04 |
| LCXNTFP1 | 14151 | 130 | 76.86 | 57422 | 238 | 50.80 | 241 | 53 | 71.10 |
| LCXNTFP2 | 4577 | 201 | 7.90 | 58025 | 182 | 25.24 | 2755 | 144 | 113.19 |
| LCXNTFP3 | 4054 | 221 | 33.35 | 91139 | 355 | 58.48 | 2532 | 180 | 50.89 |
| LCXNVZP1 | 9805 | 175 | 6.53 | 31427 | 152 | 22.41 | 3281 | 175 | 40.04 |
| LCXNVZP2 | 13385 | 374 | 90.20 | 52379 | 230 | 75.80 | 2212 | 199 | 62.97 |
| LCXNVZP3 | 7089 | 244 | 22.70 | 52205 | 245 | 24.97 | 3808 | 207 | 54.17 |
| LJAUCAMP1 | 8140 | 305 | 28.97 | 67855 | 310 | 64.96 | 2138 | 173 | 29.85 |
| LJAUCAMP2 | 11139 | 257 | 26.71 | 78416 | 336 | 72.96 | 3381 | 218 | 55.57 |
| LJAUCAMP3 | 12901 | 205 | 24.72 | 108089 | 324 | 36.87 | 3034 | 209 | 56.37 |
| LJAUIGP1 | 9726 | 327 | 50.66 | 80848 | 186 | 67.68 | 1349 | 146 | 50.66 |
| LJAUIGP2 | 2655 | 205 | 11.69 | 74211 | 81 | 15.01 | 3487 | 61 | 5.71 |
| LJAUIGP3 | 22065 | 336 | 53.10 | 54910 | 178 | 37.01 | 2592 | 121 | 13.50 |
| LJAUTFP1 | 83235 | 203 | 76.47 | 46509 | 170 | 8.77 | 2252 | 88 | 9.64 |
| LJAUTFP2 | 27262 | 231 | 65.12 | 47396 | 166 | 22.73 | 2569 | 118 | 14.25 |
| LJAUTFP3 | 13857 | 115 | 6.38 | 58238 | 81 | 13.14 | 2307 | 55 | 8.41 |
| SBCIGP1 | 16768 | 278 | 18.78 | 123546 | 75 | 20.64 | 4947 | 327 | 115.58 |
| SBCIGP2 | 11620 | 252 | 33.93 | 56161 | 83 | 5.88 | 1261 | 139 | 47.82 |
| SBCIGP3 | 7934 | 238 | 74.20 | 48758 | 96 | 9.50 | 455 | 81 | 35.26 |
| SBCTFP1 | 6418 | 262 | 24.34 | 83003 | 108 | 20.69 | 2149 | 102 | 12.74 |
| SBCTFP2 | 9905 | 273 | 39.46 | 48739 | 95 | 2.66 | 681 | 91 | 34.18 |
| SBCTFP3 | 29016 | 294 | 72.21 | 38040 | 89 | 1.82 | 811 | 97 | 33.02 |
| SBCVZP1 | 12193 | 211 | 36.23 | 38486 | 65 | 3.35 | 1092 | 91 | 22.59 |
| SBCVZP2 | 7320 | 260 | 24.82 | 52780 | 67 | 4.78 | 1089 | 73 | 15.43 |
| SBCVZP3 | 12771 | 258 | 14.91 | 44593 | 65 | 3.49 | 2585 | 79 | 9.53 |
| SCUICAMP1 | 33270 | 372 | 79.48 | 75770 | 196 | 62.74 | 818 | 149 | 46.08 |
| SCUICAMP2 | 19675 | 225 | 28.15 | 61464 | 101 | 15.17 | 268 | 24 | 34.46 |
| SCUIIGP1 | 2910 | 203 | 15.42 | 92304 | 108 | 8.40 | 2285 | 103 | 105.74 |
| SCUIIGP2 | 8002 | 138 | 12.70 | 89531 | 90 | 18.66 | 2882 | 74 | 18.13 |
| SCUIIGP3 | 57845 | 166 | 23.82 | 112637 | 79 | 18.15 | 503 | 60 | 68.27 |
| SCUITFP1 | 37423 | 273 | 54.91 | 43670 | 205 | 62.11 | 171 | 58 | 14.99 |
| SCUITFP2 | 17540 | 355 | 116.31 | 70518 | 179 | 51.96 | 356 | 92 | 104.06 |
| SCUITFP3 | 30669 | 277 | 24.96 | 82758 | 163 | 51.71 | 3710 | 218 | 57.83 |
| SCXNCAMP1 | 20325 | 394 | 86.63 | 70809 | 156 | 49.83 | 2158 | 207 | 100.33 |
| SCXNCAMP2 | 24093 | 336 | 60.91 | 68614 | 177 | 49.08 | 1315 | 168 | 4.58 |
| SCXNCAMP3 | 6097 | 121 | 10.14 | 28328 | 173 | 30.98 | 772 | 174 | 5.01 |
| SCXNIGP1 | 28357 | 356 | 54.59 | 59779 | 190 | 32.13 | 1006 | 82 | 17.95 |
| SCXNIGP2 | 20839 | 384 | 34.41 | 56928 | 171 | 34.85 | 374 | 100 | 82.37 |
| SCXNIGP3 | 21564 | 307 | 38.31 | 44027 | 107 | 33.72 | 414 | 59 | 20.57 |
| SCXNTFP1 | 11974 | 436 | 89.59 | 42962 | 212 | 68.76 | 749 | 178 | 6.76 |
| SCXNTFP2 | 29994 | 375 | 73.28 | 52009 | 225 | 66.68 | 968 | 134 | 5.27 |
| SCXNTFP3 | 12494 | 336 | 77.50 | 56929 | 250 | 66.37 | 610 | 145 | 17.92 |
| SCXNVZP1 | 1641 | 221 | 2.06 | 94653 | 146 | 23.41 | 1810 | 90 | 53.48 |
| SCXNVZP2 | 138316 | 204 | 56.90 | 56349 | 222 | 25.21 | 3720 | 81 | 53.37 |
| SCXNVZP3 | 6202 | 314 | 47.09 | 60813 | 137 | 30.37 | 829 | 52 | 72.72 |
| SJAUCAMP1 | 35415 | 416 | 112.82 | 71262 | 213 | 53.24 | 792 | 132 | 41.82 |
| SJAUCAMP2 | 47068 | 404 | 119.04 | 59086 | 202 | 86.17 | 91 | 45 | 45.69 |
| SJAUCAMP3 | 27847 | 280 | 58.88 | 78052 | 140 | 36.28 | 556 | 74 | 22.06 |
| SJAUIGP1 | 10303 | 253 | 48.90 | 69140 | 150 | 51.43 | 597 | 81 | 26.01 |
| SJAUIGP2 | 3128 | 245 | 28.45 | 43810 | 122 | 23.92 | 1470 | 50 | 3.62 |
| SJAUIGP3 | 15153 | 354 | 19.43 | 106895 | 121 | 31.13 | 383 | 76 | 48.85 |
| SJAUTFP1 | 218 | 72 | 18.18 | 66684 | 200 | 52.42 | 1520 | 208 | 77.39 |
| SJAUTFP2 | 18523 | 362 | 41.28 | 59417 | 94 | 38.07 | 395 | 93 | 59.17 |
| SJAUTFP3 | 2560 | 214 | 77.34 | 76399 | 82 | 8.26 | 1289 | 59 | 11.43 |

**Table S2.** Number and proportion (in percentages) of functional groups of OTUs considered indicators of habitat, locality and soil layer.

|  |  | **Functional group** | **ITS** | **18S** | **COI** |
| --- | --- | --- | --- | --- | --- |
| **Habitat** | **Campina** | Lichen | 1 (0.7%) | 6 (3.5%) | 0 |
|  |  | Mycorrhizae | 0 | 6 (3.5%) | 4 (1.1%) |
|  |  | Parasite | 4 (2.8%) | 9 (5%) | 31 (8.6%) |
|  |  | Phytopathogen | 16 (11%) | 22 (13%) | 9 (2.5%) |
|  |  | Saprobe | 42 (29%) | 79 (46%) | 293 (82%) |
|  |  | Unknown | 81 (56%) | 51 (29%) | 21 (5.9%) |
|  | **Igapó** | Parasite | 2 (3%) | 0 | 6 (7.6%) |
|  |  | Phytopathogen | 3 (4%) | 1 (1.6%) | 0 |
|  |  | Mycorrhizae | 0 | 13 (21%) | 2 (2.5%) |
|  |  | Saprobe | 20 (27%) | 39 (64%) | 68 (86%) |
|  |  | Unknown | 48 (66%) | 8 (13%) | 3 (3.8%) |
|  | **Terra-firme** | Mycorrhizae | 0 | 0 | 3 (2.8%) |
|  |  | Parasite | 1 (2%) | 7 (19%) | 8 (7.4%) |
|  |  | Phytopathogen | 2 (3%) | 9 (25%) | 6 (6.5%) |
|  |  | Saprobe | 34 (59%) | 15 (42%) | 87 (80%) |
|  |  | Unknown | 20 (34%) | 5 (14%) | 4 (3.7%) |
|  | **Várzea** | Mycorrhizae | 3 (2%) | 14 (14%) | 4 (2.2%) |
|  |  | Parasite | 7 (6%) | 27 (27%) | 23 (12%) |
|  |  | Phytopathogen | 3 (2%) | 7 (7%) | 1 (0.5%) |
|  |  | Saprobe | 55 (47%) | 28 (28%) | 146 (79%) |
|  |  | Unknown | 50 (42%) | 25 (25%) | 10 (5.4%) |
| **Locality** | **Benjamin Constant** | Lichen | 1 (5.7%) | 1 (1%) | 0 |
|  |  | Mycorrhizae | 3 (1.7%) | 10 (11%) | 2 (2.7%) |
|  |  | Parasite | 17 (9.8%) | 11 (12%) | 4 (5.3%) |
|  |  | Phytopathogen | 5 (2.9%) | 5 (5.5%) | 1 (1.3%) |
|  |  | Saprobe | 62 (36%) | 40 (44%) | 65 (87%) |
|  |  | Unknown | 86 (49%) | 23 (25%) | 3 (4%) |
|  | **Jaú** | Ectomycorrhiza | 1 (1.7%) | 0 | 1 (1%) |
|  |  | Mycorrhizae | 0 | 4 (5.5%) | 0 |
|  |  | Parasite | 0 | 12 (16%) | 7 (7.1%) |
|  |  | Phytopathogen | 3 (6.9%) | 2 (2.7%) | 3 (3.1%) |
|  |  | Saprobe | 17 (40%) | 28 (38%) | 84 (86%) |
|  |  | Unknown | 23 (53%) | 27 (40%) | 3 (3.1%) |
|  | **Cuieras** | Coprophilous | 1 (0.5%) | 0 | 0 |
|  |  | Endophyte | 3 (1.6%) | 0 | 0 |
|  |  | Lichen | 0 | 2 (3.4%) | 0 |
|  |  | Mycorrhizae | 13 (6.9%) | 9 (15%) | 5 (2.9%) |
|  |  | Parasite | 5 (2.6%) | 5 (8.6%) | 12 (6.9%) |
|  |  | Phytopathogen | 6 (3.2%) | 9 (15%) | 7 (4%) |
|  |  | Saprobe | 42 (22%) | 10 (17%) | 136 (79%) |
|  |  | Unknown | 129 (68%) | 23 (40%) | 13 (7.5%) |
|  | **Caxiuanã** | Mycorrhizae | 1 (1.7%) | 5 (9.6%) | 4 (2.6%) |
|  |  | Parasite | 1 (1.7%) | 0 | 10 (6.5%) |
|  |  | Phytopathogen | 1 (1.7%) | 4 (7.7%) | 3 (2%) |
|  |  | Saprobe | 22 (38%) | 10 (19%) | 126 (82%) |
|  |  | Unknown | 33 (57%) | 27 (52%) | 10 (6.5%) |
| **Soil Layla** | **Litter** | Mycorrhizae | 0 | 3 (7.1%) | 2 (1.2%) |
|  |  | Parasite | 8 (8%) | 8 (19%) | 11 (6.5%) |
|  |  | Phytopathogen | 7 (7%) | 3 (7.1%) | 6 (3.5%) |
|  |  | Saprobe | 48 (49%) | 13 (31%) | 138 (82%) |
|  |  | Unknown | 35 (36%) | 15 (36%) | 12 (7.1%) |
|  | **Soil** | Lichen | 0 | 3 (2.8%) | 0 |
|  |  | Mycorrhizae | 0 | 16 (15%) | 0 |
|  |  | Parasite | 0 | 13 (12%) | 2 (6.9%) |
|  |  | Phytopathogen | 0 | 6 (5.7%) | 0 |
|  |  | Saprobe | 5 (42%) | 39 (37%) | 24 (83%) |
|  |  | Unknown | 7 (58%) | 29 (27%) | 3 (10%) |

**Table S3.** PERMANOVA results for the community turnover. The response variables were dissimilarity matrices calculated using the Jaccard dissimilarity. In each case, the explanatory variables were the habitat type, locality, soil layer and the soil properties (physical PC1, chemical PC1, organic carbon and pH).

| **Marker** | **Predictor** | **Df** | **SumOfSqs** | **R2** | **F** | **Pr(>F)** |
| --- | --- | --- | --- | --- | --- | --- |
| **18S** | Habitat | 3 | 2.6836 | 0.12121 | 3.8829 | 0.001 |
|  | Locality | 3 | 2.2491 | 0.10158 | 3.2543 | 0.001 |
|  | Soil layer | 1 | 1.0103 | 0.04563 | 4.3856 | 0.001 |
|  | pH | 1 | 0.4348 | 0.01964 | 1.8874 | 0.005 |
|  | Carbon | 1 | 0.3659 | 0.01652 | 1.5881 | 0.016 |
|  | Chemical | 1 | 0.321 | 0.0145 | 1.3934 | 0.038 |
|  | Physical | 1 | 0.3317 | 0.01498 | 1.4399 | 0.038 |
| **COI** | Habitat | 3 | 4.795 | 0.1763 | 6.625 | 0.001 |
|  | Locality | 3 | 3.3107 | 0.12173 | 4.5742 | 0.001 |
|  | Soil layer | 1 | 1.2268 | 0.04511 | 5.085 | 0.001 |
|  | pH | 1 | 0.6467 | 0.02378 | 2.6807 | 0.001 |
|  | Carbon | 1 | 0.6097 | 0.02242 | 2.5271 | 0.001 |
|  | Chemical | 1 | 0.5144 | 0.01891 | 2.132 | 0.001 |
|  | Physical | 1 | 0.6537 | 0.02403 | 2.7095 | 0.001 |
| **ITS** | Habitat | 3 | 2.2442 | 0.07621 | 2.3657 | 0.001 |
|  | Locality | 3 | 3.2851 | 0.11156 | 3.463 | 0.001 |
|  | Soil layer | 1 | 0.9691 | 0.03291 | 3.0649 | 0.001 |
|  | pH | 1 | 0.798 | 0.0271 | 2.5236 | 0.001 |
|  | Carbon | 1 | 0.5113 | 0.01736 | 1.6169 | 0.005 |
|  | Chemical | 1 | 0.5083 | 0.01726 | 1.6075 | 0.006 |
|  | Physical | 1 | 0.8943 | 0.03037 | 2.8282 | 0.001 |

**Table S4.** Posthoc test for PERMANOVA results for the habitat and locality variables. The response variables were dissimilarity matrices calculated using the Jaccard dissimilarity. In each case, the explanatory variables were the habitat type and the locality. We used a multilevel pairwise comparison using the Permanova test. Significant values of adjust p are in bold.

| **Marker** | **Factor** | **pairs** | **Df** | **SumsOfSqs** | **F.Model** | **R2** | **p.value** | **p.adjusted** |
| --- | --- | --- | --- | --- | --- | --- | --- | --- |
| **18S** | **Habitat** | **IG_vs_TF** | **1** | **0.9392329** | **3.108242** | **0.06460934** | **0.001** | **0.006** |
|  |  | **IG_vs_VZ** | **1** | **0.6104938** | **1.99342** | **0.0553829** | **0.008** | **0.048** |
|  |  | **IG_vs_CAM** | **1** | **1.0922983** | **3.525737** | **0.08290831** | **0.001** | **0.006** |
|  |  | **TF_vs_VZ** | **1** | **0.8632262** | **2.916028** | **0.08119017** | **0.001** | **0.006** |
|  |  | **TF_vs_CAM** | **1** | **0.8100101** | **2.690887** | **0.06612997** | **0.001** | **0.006** |
|  |  | **VZ_vs_CAM** | **1** | **0.9596871** | **3.139452** | **0.1041642** | **0.001** | **0.006** |
|  | **Locality** | **BC_vs_CUI** | **1** | **1.1441614** | **3.863146** | **0.10771912** | **0.001** | **0.006** |
|  |  | **BC_vs_CXN** | **1** | **1.1528787** | **3.845809** | **0.08771213** | **0.001** | **0.006** |
|  |  | **BC_vs_JAU** | **1** | **1.0470092** | **3.595697** | **0.0956412** | **0.001** | **0.006** |
|  |  | CUI_vs_CXN | 1 | 0.6771795 | 2.141906 | 0.05335836 | 0.009 | 0.054 |
|  |  | CUI_vs_JAU | 1 | 0.4773503 | 1.53934 | 0.04589656 | 0.026 | 0.156 |
|  |  | **CXN_vs_JAU** | **1** | **0.6724604** | **2.162829** | **0.05129706** | **0.003** | **0.018** |
| **COI** | **Habitat** | **IG_vs_TF** | **1** | **1.609828** | **4.641149** | **0.09349398** | **0.001** | **0.006** |
|  |  | **IG_vs_VZ** | **1** | **1.026105** | **2.915244** | **0.07897128** | **0.001** | **0.006** |
|  |  | **IG_vs_CAM** | **1** | **1.9248** | **5.42059** | **0.12202876** | **0.001** | **0.006** |
|  |  | **TF_vs_VZ** | **1** | **1.318142** | **4.007158** | **0.10828062** | **0.001** | **0.006** |
|  |  | **TF_vs_CAM** | **1** | **1.545337** | **4.610601** | **0.10820315** | **0.001** | **0.006** |
|  |  | **VZ_vs_CAM** | **1** | **1.615844** | **4.796855** | **0.15085942** | **0.001** | **0.006** |
|  | **Locality** | **BC_vs_CUI** | **1** | **1.48318** | **4.225178** | **0.11663651** | **0.001** | **0.006** |
|  |  | **BC_vs_CXN** | **1** | **1.574299** | **4.582021** | **0.10277732** | **0.001** | **0.006** |
|  |  | **BC_vs_JAU** | **1** | **1.230438** | **3.50392** | **0.0934281** | **0.001** | **0.006** |
|  |  | **CUI_vs_CXN** | **1** | **1.108926** | **3.083491** | **0.07505427** | **0.001** | **0.006** |
|  |  | CUI_vs_JAU | 1 | 0.727061 | 1.961344 | 0.05775226 | 0.014 | 0.084 |
|  |  | **CXN_vs_JAU** | **1** | **1.139812** | **3.172215** | **0.07347816** | **0.001** | **0.006** |
| **ITS** | **Habitat** | IG_vs_TF | 1 | 0.6299698 | 1.534165 | 0.03296857 | 0.014 | 0.084 |
|  |  | **IG_vs_VZ** | **1** | **0.710604** | **1.712124** | **0.04794237** | **0.003** | **0.018** |
|  |  | IG_vs_CAM | 1 | 0.6651875 | 1.622503 | 0.03994099 | 0.007 | 0.042 |
|  |  | TF_vs_VZ | 1 | 0.6508677 | 1.663098 | 0.04797892 | 0.018 | 0.108 |
|  |  | **TF_vs_CAM** | **1** | **0.7560525** | **1.942205** | **0.04862539** | **0.001** | **0.006** |
|  |  | VZ_vs_CAM | 1 | 0.7338455 | 1.900483 | 0.06575956 | 0.002 | 0.012 |
|  | **Locality** | **BC_vs_CUI** | **1** | **1.1839634** | **3.34367** | **0.09460449** | **0.001** | **0.006** |
|  |  | **BC_vs_CXN** | **1** | **1.3424566** | **3.445567** | **0.07930767** | **0.001** | **0.006** |
|  |  | **BC_vs_JAU** | **1** | **1.4768756** | **3.985484** | **0.10492125** | **0.001** | **0.006** |
|  |  | CUI_vs_CXN | 1 | 0.6270052 | 1.563045 | 0.03950769 | 0.017 | 0.102 |
|  |  | **CUI_vs_JAU** | **1** | **0.9382049** | **2.449247** | **0.07109724** | **0.001** | **0.006** |
|  |  | **CXN_vs_JAU** | **1** | **0.7295278** | **1.767299** | **0.04231297** | **0.003** | **0.018** |

**Table S5.** Number and proportion (in percentages) of functional groups of OTUs by habitat type.

|  |  | **Functional group** | **ITS** | **18S** | **COI** |
| --- | --- | --- | --- | --- | --- |
| **Habitat** | **Campina** | Lichen | 13 (1%) | 23 (2%) | 0 |
|  |  | Mycorrhizae | 26 (2%) | 91 (9%) | 20 (2%) |
|  |  | Parasite | 34 (3%) | 111 (11%) | 53 (5%) |
|  |  | Phytopathogen | 83 (7%) | 53 (5%) | 23 (2%) |
|  |  | Saprobe | 367 (32%) | 353 (34%) | 842 (82%) |
|  |  | Unknown | 615 (54%) | 407 (39%) | 92 (9%) |
|  | **Igapó** | Lichen | 6 (0.6%) | 22 (2%) | 0 |
|  |  | Mycorrhizae | 41 (4%) | 81 (8%) | 22 (4%) |
|  |  | Parasite | 39 (4%) | 92 (9%) | 35 (6%) |
|  |  | Phytopathogen | 33 (3%) | 64 (7%) | 7 (1%) |
|  |  | Saprobe | 322 (33%) | 353 (36%) | 480 (81%) |
|  |  | Unknown | 542 (55%) | 356 (37%) | 48 (8%) |
|  | **Terra-firme** | Lichen | 3 (0.3%) | 17 (2%) | 0 |
|  |  | Mycorrhizae | 43 (4%) | 89 (10%) | 26 (3%) |
|  |  | Parasite | 56 (5%) | 74 (9%) | 54 (7%) |
|  |  | Phytopathogen | 38 (3%) | 52 (6%) | 12 (1%) |
|  |  | Saprobe | 495 (44%) | 294 (34%) | 656 (81%) |
|  |  | Unknown | 495 (44%) | 335 (39%) | 60 (7%) |
|  | **Várzea** | Lichen | 1 (0.2%) | 15 (2%) | 0 |
|  |  | Mycorrhizae | 8 (1%) | 65 (9%) | 11 (2%) |
|  |  | Parasite | 27 (5%) | 63 (9%) | 35 (7%) |
|  |  | Phytopathogen | 22 (4%) | 48 (7%) | 7 (1%) |
|  |  | Saprobe | 261 (47%) | 251 (35%) | 416 (82%) |
|  |  | Unknown | 238 (43%) | 281 (39%) | 40 (8%) |


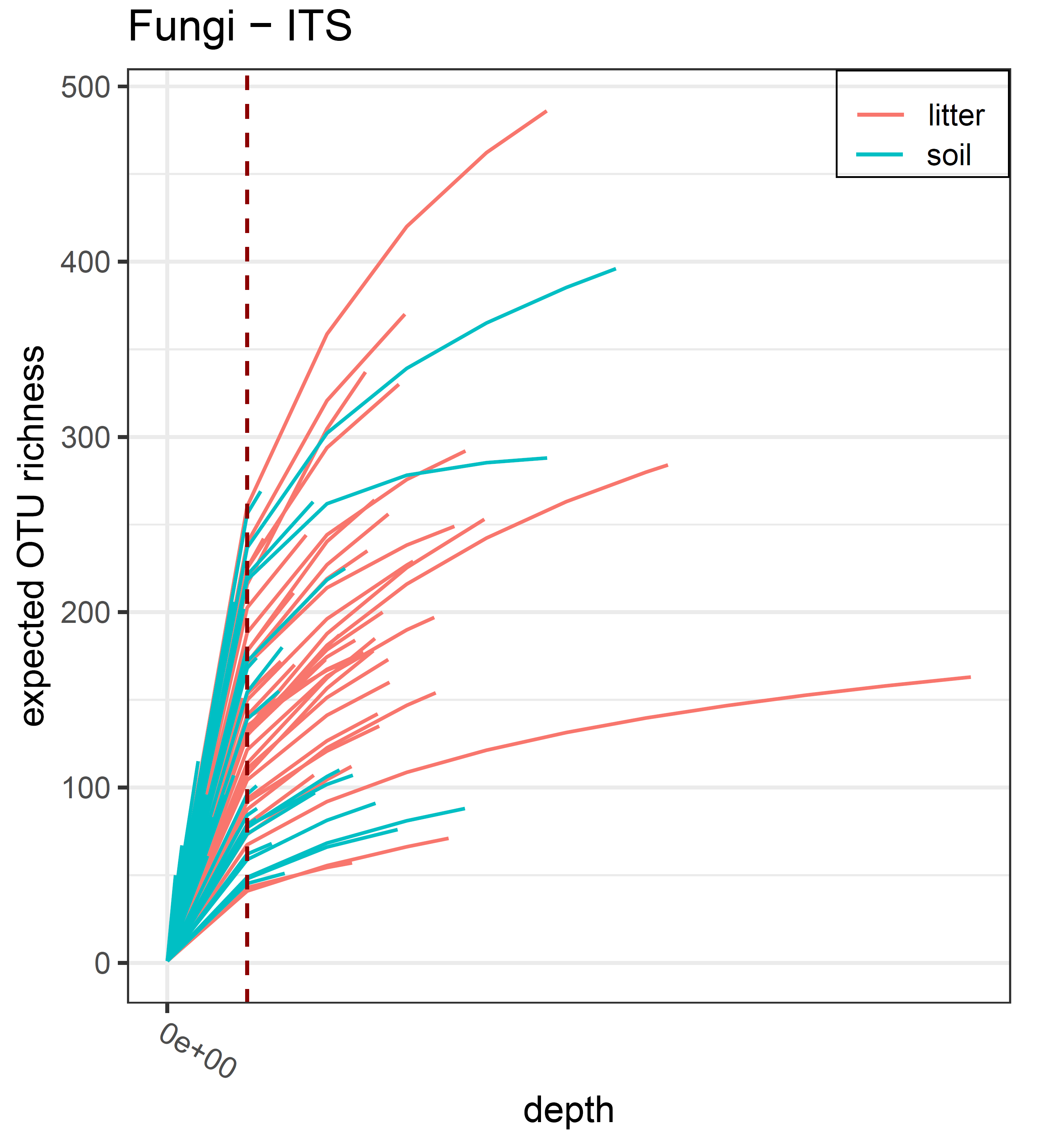


**Figure S1** - **Rarefaction curves.** Rarefaction by sample for long read ITS data. The red dashed line shows the minimum number of reads. The different colours represent the different sample type: red are litter samples and blue are the soil samples. The soil litter samples are closer to the asymptote. However, the soil samples are not close to the asymptote.
